# Autonomic-salience stability as a candidate Gate for awake low-dose ketamine: a systems neuroscience framework with a clinical anchor

**DOI:** 10.3389/fnsys.2026.1880737

**Published:** 2026-07-01

**Authors:** Kei Torii, Maho Jinno

**Affiliations:** 1 Tokyo Anesthesiology Clinic, Tokyo, Japan; 2 Nagoya Anesthesiology Clinic, Medical Corporation Shinkakai, Nagoya, Japan

**Keywords:** autonomic gating, central autonomic network, chronic pain, frontoparietal network, heart rate variability, ketamine, salience network, thalamo-cortical gating

## Abstract

Ketamine responses vary across patients and sessions, suggesting that dose alone is an incomplete organizing principle. We propose a state-first Gate-Amplifier-Reintegration framework in which awake low-dose ketamine acts primarily as an Amplifier of transient network flexibility, whereas autonomic-salience stability is treated as a candidate Gate that may shape whether this flexibility remains steerable. In this framework, cardio-autonomic and interoceptive state may constrain or modulate salience-network gain, interoceptive precision, and thalamocortical selectivity, thereby influencing whether ketamine-associated loosening of default-mode constraints is available for frontoparietal-control-compatible reintegration or drifts toward dysphoric dissociation and vigilance instability. We formalize a three-step sequence: Gate, Amplifier, Reintegration. Gate refers to candidate autonomic-salience stability; Amplifier refers to awake low-dose ketamine delivered under operational invariants that preserve vigilance and behavioral interpretability; Reintegration refers to the organization of ketamine-amplified flexibility into language, joint attention, task context, and action-oriented consolidation. Heart-rate variability (HRV) is used only as a bounded peripheral state-verification proxy. We distinguish observed Autonomic Affirmative Window quality assurance (AAW-QA), a post-sequence quality-assurance signal, from AAW-Gate, a proposed prospective pre-dose criterion. The framework is informed by, but not validated by, observations from a single-center outpatient chronic pain care pathway using awake low-dose ketamine and route-defined cervicothoracic sympathetic modulation. These observations document clinical provenance but do not provide comparative efficacy evidence, causal efficacy, dose-sparing evidence, salience-network mediation, or HRV biomarker validity. The clinical provenance was nonrandomized, chart-based, clinician-directed, and lacked concurrent neural measurement. The framework yields falsifiable predictions: prospective Gate manipulation should reproducibly alter pre-dose autonomic state; prospectively defined AAW-Gate-positive sessions should be tested for convergence with low-burden EEG/fNIRS markers of salience switching and task control; and randomized Gate designs should determine whether autonomic shifts moderate, and in adequately powered designs mediate, session-level tolerability, reintegration, and usable clinical change. Alternative accounts, including analgesia, expectancy, clinician attention, workflow era, documentation bias, respiratory/postural effects, and photoplethysmography (PPG) artifact, are treated as competing explanations that future designs must separate.

## Introduction: Why a Gate-Amplifier-Reintegration framework is needed

Ketamine responses vary across patients, sessions, indications, and clinical settings. This variability is often approached through a dose-first lens: if the response is insufficient, the dose, duration, route, or repetition schedule is reconsidered. Dose is undeniably important, but it is an incomplete organizing principle. A ketamine session is not only a pharmacologic exposure. It is also a state transition occurring in a patient with a pre-existing configuration of pain, vigilance, interoception, autonomic tone, attention, expectation, dissociation vulnerability, and capacity for reintegration.

This distinction matters because ketamine does not simply produce a uniform therapeutic state. Subanesthetic ketamine has been associated with alterations in large-scale functional connectivity involving default-mode, salience, executive-control, thalamo-cortical, and prefrontal systems (Mueller et al., 2018; Fleming et al., 2019; Höflich et al., 2015; Zacharias et al., 2020; Moujaes et al., 2024). These network effects are not isolated from vigilance, arousal, or individual variability. A dose-first view may describe exposure, but it does not fully explain why one session becomes clinically steerable while another becomes dysphoric, disorganized, sedating, or difficult to integrate.

We call this the Gate-Amplifier-Reintegration framework. The candidate Gate is autonomic-salience stability; the Amplifier is awake low-dose ketamine; and Reintegration is the organization of ketamine-amplified flexibility into language, joint attention, task context, decisions, and next-day action. The framework is state-first because it treats the pre-ketamine autonomic, interoceptive, attentional, and vigilance state as a condition that may shape how ketamine-induced flexibility is used. By Amplifier, we mean ketamine’s capacity to transiently increase network flexibility, loosen high-level constraints, and create a window in which entrenched predictions or defensive salience assignments can be updated. By Gate, we mean the upstream state condition that may influence whether this flexibility becomes steerable, dysphoric, disorganized, sedating, or difficult to integrate.

The salience network provides the systems-neuroscience bridge for this framework. Salience-network hubs, including anterior insula and anterior cingulate regions, have been implicated in detecting salient internal and external events and in facilitating switching between internally oriented default-mode activity and externally or task-oriented executive/frontoparietal control (Seeley et al., 2007; Sridharan et al., 2008; Menon and Uddin, 2010). This switching role is clinically relevant because ketamine-induced flexibility is not automatically therapeutic. If salience switching is unstable or threat-biased, ketamine may amplify dysphoric dissociation, bodily alarm, or vigilance lability. If switching remains stable and controllable, the same pharmacologic flexibility may be more likely to support frontoparietal-control-compatible reintegration.

Autonomic and interoceptive state may constrain this switching process. The central autonomic network and interoceptive systems overlap with insular and cingulate regions involved in salience processing (Beissner et al., 2013; Ferraro et al., 2022; Critchley et al., 2004). Neurovisceral integration accounts further link autonomic regulation with attention, emotion regulation, and prefrontal control (Thayer and Lane, 2000). Heart-rate variability (HRV) refers to beat-to-beat variation in cardiac intervals and is commonly used as a peripheral index of cardio-autonomic regulation. Because central autonomic network regions participate in autonomic control, HRV may provide a limited peripheral process readout of autonomic state; however, it does not measure central autonomic network activity or salience-network activity directly.

Chronic pain is commonly defined as pain that persists or recurs for longer than 3 months and is increasingly understood as involving sensory, affective, cognitive, interoceptive, and autonomic dimensions rather than nociception alone (Treede et al., 2015; Raja et al., 2020). Chronic pain is therefore a particularly relevant clinical anchor for this framework. Chronic pain is not only persistent nociception or peripheral sensitization; it also involves altered salience, interoception, threat appraisal, affective regulation, autonomic tone, and functional connectivity across default-mode and salience networks (Borsook et al., 2013; van Ettinger-Veenstra et al., 2019; Johansson et al., 2024). A state-first ketamine framework is therefore especially relevant in chronic pain because pain is both a sensory input and a body-state interpretation that can dominate salience allocation before ketamine is introduced.

The clinical observation motivating this article comes from a single-center outpatient chronic pain care pathway in which awake low-dose ketamine was delivered under operational invariants, and routine high-thoracic/low-cervical epidural sympathetic modulation could be timed before ketamine when clinically indicated. In that pathway, route-defined patterns of chart-documented improvement, observed ketamine dose, patient-level safety monitoring, and post-sequence HRV quality assurance raised the question of whether autonomic state should be measured and manipulated prospectively before ketamine. These observations are not treated as proof of efficacy, dose-sparing, safety generalizability, salience-network mediation, or HRV biomarker validity. They are treated as observational provenance for a falsifiable framework.

This article therefore has two aims. First, we formalize the Gate-Amplifier-Reintegration framework of ketamine response. In this framework, autonomic-salience stability functions as a candidate Gate, ketamine functions as the Amplifier, and Reintegration helps determine whether amplified flexibility becomes usable change. Second, we define falsifiable predictions and low-burden physiological and behavioral assays (assay-lite tests): prospective Gate manipulation, pre-dose AAW-Gate measurement, EEG/fNIRS or behavioral markers of salience switching and task-control engagement, reintegration outputs, and explicit alternative explanations that would revise or falsify the framework. The goal is not to prove a clinical intervention retrospectively. The goal is to convert a clinical observation into a testable systems-neuroscience program.

## Core claim: ketamine is the Amplifier, not the Gate

The central claim is deliberately simple: ketamine is not the Gate; ketamine is the Amplifier. The candidate Gate is autonomic-salience stability. Ketamine may transiently increase network flexibility, loosen high-level constraints, and alter large-scale connectivity, but the direction and usability of that flexibility may depend on the state into which the drug is introduced.

This distinction is necessary because ketamine does not act in an empty system. It acts on a patient whose salience, interoceptive, autonomic, attentional, and vigilance states are already configured before the infusion begins. In this view, the question is not simply whether ketamine loosens default-mode constraints. The more clinically relevant question is whether the patient’s state allows that loosening to be routed toward controlled reintegration rather than dysphoric dissociation, defensive monitoring, or vigilance instability.

Ketamine network studies support the view that subanesthetic ketamine can alter large-scale functional connectivity rather than producing a single local effect. Pharmacological fMRI studies have reported ketamine-related changes involving the default mode network, salience network, executive-control networks, thalamic connectivity, and dorsolateral prefrontal regions (Mueller et al., 2018; Fleming et al., 2019; Höflich et al., 2015). Simultaneous fMRI/EEG work further suggests that ketamine-induced connectivity changes are entangled with changes in vigilance, meaning that network effects cannot be cleanly interpreted without attention to state (Zacharias et al., 2020). More recent work also emphasizes substantial inter-individual variability in ketamine-induced whole-brain connectivity signatures (Moujaes et al., 2024). These findings do not prove the Amplifier component, but they make a dose-first view insufficient.

The Gate is therefore not a drug dose, a subjective intensity target, or a procedural label. It is a state condition. A candidate Gate-positive session is one in which autonomic and attentional conditions are stable enough for salience-mediated switching to remain usable while ketamine increases flexibility. A candidate Gate-negative session is one in which the same pharmacologic amplification may increase noise: bodily threat signals, dysphoric dissociation, autonomic alarm, unstable vigilance, or unstructured affective cycling.

This is why autonomic state matters. The central autonomic network and interoceptive systems overlap anatomically and functionally with regions implicated in salience processing, including insular and cingulate cortices (Beissner et al., 2013; Ferraro et al., 2022; Critchley et al., 2004). Neurovisceral integration models further link autonomic regulation, affective regulation, attentional control, and adaptive behavior (Thayer and Lane, 2000). The claim is not that peripheral autonomic measures directly read out salience-network activity. They do not. The more defensible claim is that autonomic state may set boundary conditions for salience-network gain, interoceptive precision, and thalamo-cortical selectivity.

The clinical implication is that ketamine sessions should not be optimized only by increasing dose or pursuing subjective intensity. A more useful target may be steerable flexibility: a state in which the patient remains awake, physiologically stable, linguistically accessible, and capable of converting altered experience into frontoparietal-control-compatible reintegration. In this framework, low-dose awake ketamine is valuable not because it is weak, but because it preserves the control systems required to organize the altered state. The therapeutic aim is not maximal dissociation. The aim is guided loosening followed by usable reorganization.

This reframing also changes how cervicothoracic sympathetic modulation should be interpreted. The hypothesis is not that an epidural block directly activates the salience network or mechanically potentiates ketamine. Rather, cervicothoracic sympathetic modulation is one candidate clinical lever for shifting autonomic boundary conditions before ketamine exposure. If it reduces sympathetic noise, stabilizes interoceptive state, or lowers defensive vigilance, it may improve the Gate through which ketamine amplification occurs. Whether this is true requires prospective testing. The clinical cohort serves only as an anchor for this hypothesis, not as proof.

**FIGURE 1:**
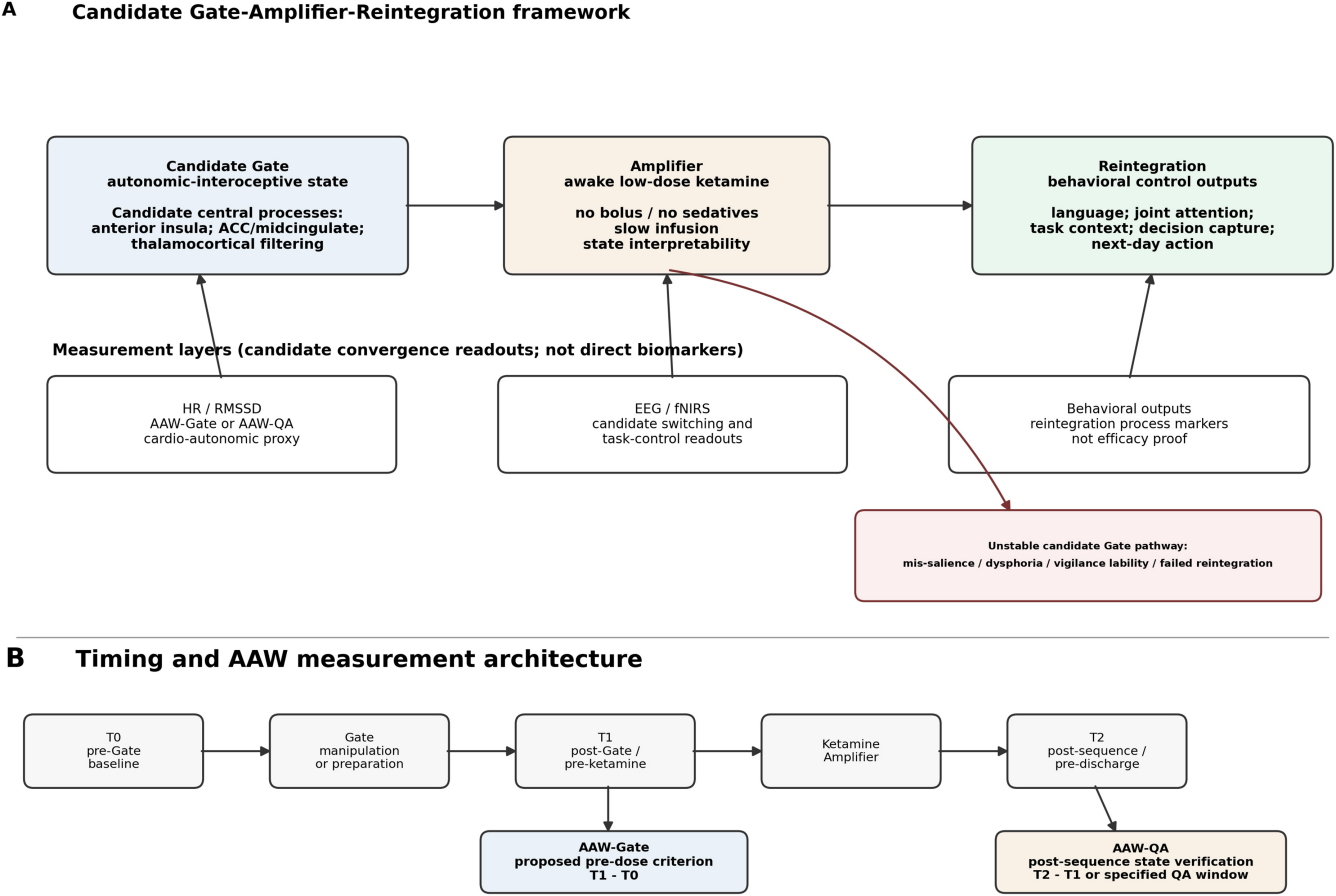
Candidate Gate-Amplifier-Reintegration framework and measurement architecture. **(A)** Autonomic-salience stability is framed as a candidate Gate that may set boundary conditions for ketamine amplification and behavioral reintegration. Candidate central nodes and processes include anterior insula, anterior cingulate/midcingulate cortex, and thalamocortical filtering; these are theoretical targets, not directly measured in the clinical anchor. **(B)** AAW-Gate is a proposed prospective pre-dose criterion, whereas AAW-QA is a post-sequence quality-assurance readout. HR/RMSSD, EEG/fNIRS, and behavioral outputs are convergence readouts rather than biomarkers. Evidence status differs across the diagram: network relationships are literature-derived, the clinical pathway is observational provenance, and the assay layers are future tests.

Figure 1 summarizes the candidate Gate-Amplifier-Reintegration framework and the associated measurement architecture. Boxes 1–3 summarize the operational definitions, Amplifier invariants, and HRV/AAW terminology and interpretive boundaries used throughout the framework.

BOX 1 **Operational definitions and measurement strategy for the Gate-Amplifier-Reintegration framework.**

Autonomic-salience stability. A session-level candidate state in which autonomic, interoceptive, attentional, and vigilance conditions are sufficiently organized for salience-mediated switching to remain usable during ketamine amplification. It is not a directly observed neural state and is not equivalent to HRV alone. It should be assessed through convergent physiological, behavioral, and protocol-quality indicators.

Candidate Gate-positive session. A prospective, pre-dose session-level classification to be tested in future studies. A session would be considered candidate Gate-positive only when minimum measurement invariants are satisfied, a pre-dose HR-RMSSD directional concordance pattern is present, major protocol deviations are absent, and the patient remains awake, verbally accessible, and physiologically stable. This classification is provisional and does not establish mechanism, treatment readiness, or clinical success.

Candidate Gate-negative session. A session in which pre-dose autonomic, vigilance, respiratory, behavioral, or protocol conditions suggest unstable state entry. Examples include absent HR-RMSSD concordance, high defensive vigilance, dysphoria, respiratory instability, major posture or oxygen changes, excessive movement artifact, or inability to participate in orienting cues. Gate-negative status does not mean treatment failure; it means the session is not a clean test of the proposed Gate sequence.

Steerable flexibility. A ketamine-amplified state in which the patient remains awake, physiologically stable, verbally accessible, and able to respond to simple orienting or reintegration cues. It is distinct from subjective intensity, dissociation depth, or sedation. Reintegration. The process by which ketamine-amplified flexibility is organized into language, joint attention, task context, decision, movement, written plan, or next-day action. Reintegration outputs are behavioral process markers, not direct measurements of frontoparietal network activity and not substitutes for validated clinical outcomes.

Minimum timing structure for prospective tests. Prospective studies should distinguish at least four windows: pre-Gate baseline, post-Gate/pre-ketamine, ketamine exposure, and post-ketamine reintegration. This timing separates AAW-Gate from AAW-QA and prevents post-sequence HRV changes from being misread as pre-dose Gate evidence.

Minimum physiological requirements. Same posture, same device and sensor placement, comparable window length, beat-to-beat signal quality control, artifact screening, and documentation of respiration, oxygen use, medication changes, nausea, movement, conversation, environmental disruption, and protocol deviations.

Threshold status. At present, the only proposed physiological threshold is directional rather than magnitude-based: heart rate decrease and RMSSD increase within the specified interval. Magnitude thresholds, behavioral cutoffs, and clinically useful decision thresholds are not established by the present clinical anchor and should be estimated prospectively.

BOX 2 **AAW terminology and interpretive boundaries.**

• AAW definition. AAW = delta HR < 0 and delta RMSSD > 0 within a specified measurement interval.

• What AAW is.

• A directional HRV-based state-verification pattern.

• A session-level signal, not a patient trait.

• A bounded proxy for cardio-autonomic movement in the predicted Gate direction.

• A quality-assurance signal when measured after the workflow.

• A candidate prospective Gate criterion when measured before ketamine. What AAW is not.

• Not a diagnostic test.

• Not a biomarker of salience-network activation.

• Not a measure of vagal dominance or central autonomic network engagement.

• Not proof of ketamine efficacy, treatment readiness, or clinical success.

• Not a surrogate for validated patient-reported outcomes.

• Not a stand-alone treatment decision rule. AAW-QA. A post-sequence or retrospective quality-assurance readout. It asks whether the completed workflow was associated with the predicted HRV direction. AAW-Gate. A prospective pre-dose criterion to be tested before ketamine exposure. It asks whether the patient has entered a state compatible with Gate readiness. Minimum measurement invariants.

• Same posture across windows.

• Same device and sensor placement.

• Comparable window length.

• Beat-to-beat signal quality control.

• Artifact screening.

• Documentation of respiration, oxygen use, medication changes, nausea, movement, conversation, environmental disruption, and protocol deviations.

Interpretive rule. Showing the AAW pattern does not mean mechanism proven. Absence of the AAW pattern does not mean session failed. AAW is meaningful only when interpreted alongside behavior, tolerability, reintegration, and prospective test design.

BOX 3 **Operational invariants for the Amplifier step.**

Purpose. To preserve interpretability of ketamine as an Amplifier of network flexibility rather than an uncontrolled pharmacologic perturbation. Core invariants.

• Awake state: patient remains verbally accessible and able to participate in orienting or reintegration cues.

• No sedative premedication: avoids confounding of vigilance, respiratory pattern, autonomic tone, memory, dissociation, and language availability.

• No ketamine bolus: avoids abrupt state transitions and dissociative peaks that may mimic Gate instability.

• Slow continuous infusion: allows gradual amplification of a prepared state and observation of steerability.

• Low-dose strategy: dose is not escalated to chase subjective intensity; the target is usable flexibility.

• Stable posture: supine or otherwise fixed posture across measurement windows to preserve HRV interpretability.

• Room air unless clinically indicated: oxygen use is allowed when needed but should be documented because respiratory/oxygen changes affect physiological interpretation.

• Avoid mid-infusion dose escalation: maintains distinction between state quality and pharmacologic intensity.

• Quiet, predictable environment: reduces uncontrolled salience capture.

• Continuous monitoring: HR, SpO2, blood pressure checks, nausea/vertigo assessment, verbal coherence, and discharge readiness.

• Deviation logging: sedatives, boluses, posture changes, oxygen use, environmental disruption, dose changes, dysphoria, desaturation, nausea, or reintegration failure are recorded as interpretive boundary events.

Interpretive rule. A session that violates these invariants may still be clinically valid, but it should not be treated as a clean test of the Gate-Amplifier-Reintegration sequence.

## The Gate: autonomic state may constrain or modulate salience switching

The Gate in this framework is not an anatomical site and not a procedural label. It is a state condition: the degree to which autonomic, interoceptive, attentional, and vigilance systems are organized enough for salience-mediated switching to remain stable during ketamine amplification.

The salience network is positioned to integrate internal bodily signals, affective salience, external sensory events, and task demands. Its core nodes, including the anterior insula and anterior cingulate cortex, overlap conceptually and anatomically with systems implicated in interoception, autonomic regulation, and action readiness. The salience network has been described as distinct from executive-control networks, and the right fronto-insular cortex has been implicated in switching between default-mode and central-executive networks (Seeley et al., 2007; Sridharan et al., 2008; Menon and Uddin, 2010). This switching role is the key reason the salience network is central to the present framework.

However, salience switching does not occur in a vacuum. A patient enters a ketamine session with a pre-existing body state: pain, sympathetic tone, respiratory pattern, nausea, fear, vigilance, expectation, fatigue, medication load, and interoceptive uncertainty. These variables are often treated clinically as background noise. In a Gate-Amplifier-Reintegration framework, they are not background. They are part of the control architecture.

The central autonomic network provides a neuroanatomical and functional bridge for this claim. Neuroimaging meta-analyses of autonomic processing implicate a distributed set of regions, including insular and cingulate cortices, in central autonomic regulation (Beissner et al., 2013; Ferraro et al., 2022). This does not mean that peripheral HRV measures central autonomic network activity. It means that peripheral cardio-autonomic readouts can be used as limited process signals when interpreted alongside posture, respiration, artifact control, behavior, and cortical convergence tests.

The Gate should therefore be understood as a boundary-condition model. Autonomic state may influence salience switching through at least four interacting routes: gain, threshold, interoceptive precision, and thalamo-cortical selectivity. When sympathetic alarm, pain threat, nausea, or respiratory instability is high, internally generated bodily signals may become disproportionately salient. Under ketamine, amplification of such signals may increase dysphoric dissociation or defensive monitoring rather than therapeutic flexibility. Conversely, when autonomic noise is reduced, salience detection may become less threat-biased and more available for task-relevant switching.

A high-vigilance state may lower the threshold for threat salience, causing benign sensory or interoceptive events to be interpreted as urgent. Predictive and inferential accounts of interoception further describe bodily feeling states as actively inferred from interoceptive signals and their predicted causes (Seth, 2013; Seth and Friston, 2016). If interoceptive precision becomes excessive or unstable, ketamine may amplify bodily uncertainty. If interoceptive precision becomes more regulated, the same pharmacologic flexibility may become easier to integrate.

Ketamine can also alter thalamo-cortical connectivity and large-scale network organization (Höflich et al., 2015). A Gate-Amplifier-Reintegration framework predicts that the functional meaning of this alteration depends on whether the system is already biased toward defensive monitoring or toward controlled exploration. Thalamo-cortical loosening is not inherently therapeutic. It becomes useful only when salience filtering and frontoparietal control can still organize the altered input.

This is the rationale for considering cervicothoracic sympathetic modulation as a candidate Gate lever. The hypothesis is not that a high-thoracic or low-cervical epidural block directly stimulates the salience network. The more defensible hypothesis is that reducing sympathetic load and stabilizing cardio-autonomic/interoceptive state may improve the boundary conditions under which salience-mediated switching occurs. If the Gate is stabilized, ketamine amplification may be more likely to lead to reintegration. If the Gate is unstable, ketamine amplification may instead magnify dysphoric dissociation, vigilance lability, or somatic threat.

This distinction prevents the framework from becoming a simple procedural claim. “Epidural before ketamine” is not the theory. “Autonomic-salience stability before ketamine amplification” is the theory. Cervicothoracic sympathetic modulation is one candidate method of testing that theory in a specific clinical setting. Other methods - breathing regulation, posture stabilization, environmental control, non-invasive autonomic modulation, or pharmacologic autonomic stabilization - could in principle test the same Gate concept, provided they are operationalized prospectively and evaluated with physiological and cortical readouts.

## The Amplifier: awake low-dose ketamine under operational invariants

In the Gate-Amplifier-Reintegration framework, ketamine is treated as an Amplifier only under conditions that preserve interpretability and standard ketamine monitoring principles (Cohen et al., 2018; Schwenk et al., 2018). If a session includes sedative premedication, bolus dosing, posture shifts, oxygen changes unrelated to clinical need, mid-infusion dose escalation, uncontrolled sensory stimulation, or inconsistent monitoring, then observed changes in behavior, HRV, dissociation, or tolerability become difficult to attribute. The session may still be clinically reasonable, but it is no longer a clean test of the Gate-Amplifier-Reintegration sequence.

The Amplifier step therefore requires operational invariants. These invariants are not proposed as universal rules for all ketamine practice. They are constraints for a specific Gate-Amplifier-Reintegration framework in which ketamine’s role is to increase network flexibility while preserving enough wakefulness, language, and frontoparietal control for reintegration. In this setting, low-dose ketamine is not weak ketamine. It is ketamine delivered in a range and manner intended to preserve steerability.

Wakefulness is the first invariant. The patient should remain accessible to verbal contact, able to report subjective state, and able to participate in simple orienting or reintegration cues. This is not merely a safety preference. It is part of the theory. If the patient cannot participate, frontoparietal-control-compatible reintegration cannot be assessed. Similarly, sedative premedication is avoided in this model because it can alter vigilance, respiratory pattern, autonomic tone, memory, language availability, and dissociative experience.

The second invariant is no ketamine bolus. Bolus dosing can create abrupt state transitions, rapid changes in arousal, and transient dissociative peaks. Those peaks may be difficult to distinguish from Gate instability. A slow continuous infusion better matches the framework: the Amplifier is introduced gradually into a prepared state, allowing the clinician to observe whether the patient remains physiologically stable, verbally accessible, and behaviorally steerable.

The third invariant is low-dose delivery without chasing intensity. In this framework, dose is not the primary target. The target is usable flexibility. Escalating dose to pursue a stronger subjective experience risks converting a controlled Amplifier into a destabilizing perturbation. This is especially important because ketamine-induced changes in functional connectivity are state-dependent and can be entangled with changes in vigilance (Zacharias et al., 2020). A dose-first approach may obscure whether the observed session state reflects therapeutic flexibility, sedation, dysphoria, vigilance loss, or nonspecific intensity.

Other invariants protect physiological interpretation: stable posture, preferably supine; room-air breathing unless oxygen is clinically indicated; a quiet and predictable environment; continuous monitoring; and explicit deviation logging. These are not only clinical procedures. They are epistemic procedures. They protect the meaning of physiological readouts and distinguish state quality from pharmacologic intensity, environmental disruption, respiratory change, or rescue interventions.

This point also reframes dose. In a retrospective clinical anchor, observed ketamine dose is clinician-selected and cannot be interpreted as a biological requirement. Lower observed dose in one workflow does not prove dose-sparing efficacy. It may reflect clinician judgment, patient selection, route selection, calendar era, pain phenotype, or workflow stability. In the present framework, dose is interpreted cautiously: not as proof that the Gate reduces pharmacologic requirement, but as a process variable that may become meaningful only in prospective randomized Gate designs.

## Reintegration: organizing flexibility into action

The Gate-Amplifier sequence is incomplete without Reintegration. A stable Gate may reduce autonomic noise, and ketamine may amplify network flexibility, but neither process by itself guarantees usable clinical change. A ketamine session can be vivid, emotionally intense, or subjectively meaningful and still fail to produce a coherent decision, behavior, or next-day action. In the Gate-Amplifier-Reintegration framework, the therapeutic target is therefore not altered experience alone. The target is altered experience that can be organized into language, joint attention, task context, and action.

Reintegration is the process by which ketamine-amplified flexibility is routed into frontoparietal control and goal-directed behavior. This is why wakefulness and low-dose delivery matter. If the patient is too sedated, too disorganized, or too dysphoric to participate, the session may remain pharmacologically active but become clinically less steerable. The altered state may be experienced, but not organized. It may be remembered as intense, but not converted into a usable plan.

In network terms, Reintegration depends on preserving access to systems that support cognitive control, task context, and flexible implementation of goals. The frontoparietal control network has been described as a flexible hub system that can alter its connectivity with other networks across task states and support adaptive task control (Cole et al., 2013; Zanto and Gazzaley, 2013). Fronto-parietal representations of task context are also implicated in aligning perception, attention, and response selection with internal goals (Waskom et al., 2014). These concepts map directly onto the clinical problem: after ketamine loosens rigid interpretations or default-mode constraints, the patient must still be able to organize the altered material into a goal-relevant form.

Reintegration is therefore not a decorative post-session conversation. It is part of the proposed control sequence. If the Gate prepares the body-state conditions and the Amplifier opens flexibility, Reintegration helps determine whether that flexibility is captured by frontoparietal control or dissipates into unstructured experience. The clinician’s task is not to force interpretation, but to provide enough language, joint attention, and task structure for the patient to convert the session into something that can be carried forward.

Language is central because it compresses diffuse bodily, affective, and perceptual change into shareable units (Fedorenko and Thompson-Schill, 2014). Joint attention is equally important. By joint attention, we mean coordinated patient-clinician attention toward a shared target, such as a bodily sensation, movement, sentence, decision, or action plan. Ketamine sessions can become internally absorbed; that inward shift may be useful, but it can also detach the patient from shared reality testing and task direction. Joint attention provides an interpersonal anchor: patient and clinician coordinate attention toward a bodily state, a movement, a sentence, a decision, or a next action (Mundy and Newell, 2007; Caruana et al., 2015). Brief tasks serve the same purpose. A task may be as simple as naming the dominant bodily change, comparing two pain-related movements, selecting one next-day behavior, writing a sentence, or identifying a decision that now feels possible.

This also reframes outcome measurement. In the Gate-Amplifier-Reintegration framework, the most meaningful near-term session outcome may not be a global symptom rating. It may be a usable change output: a coherent statement, a movement attempted, a decision made, a message sent, a plan written, a feared action reclassified, or a next-day behavior captured. Such outputs are not substitutes for validated patient-reported outcomes in clinical trials. They are process markers. They indicate whether the ketamine-amplified state was organized into goal-directed control rather than remaining an unstructured subjective event. These outputs are not assumed to measure frontoparietal network activity directly; rather, they are behavioral control readouts that operationalize the availability of task context, shared attention, and goal-directed organization during or after ketamine-associated state loosening.

The distinction between intensity and steerability is critical. A high-intensity session can fail if it produces dysphoria, fragmentation, or inability to act. A low-intensity session can succeed if it produces a clear action, reduced defensive salience, or a stable change in how pain is interpreted. The aim is not maximal dissociation, maximal affect, or maximal phenomenological novelty. The aim is steerable change.

## HR-RMSSD directional concordance as a bounded process-quality signal

We use Autonomic Affirmative Window (AAW) as shorthand for a bounded heart rate/root mean square of successive differences (HR-RMSSD) directional concordance pattern, not as a neural biomarker or normative label of clinical readiness. It is defined directionally as a paired short-window pattern: heart rate decreases and RMSSD increases under standardized posture, sensor, timing, and artifact-control conditions. In shorthand, AAW = delta HR < 0 and delta RMSSD > 0.

This definition is intentionally modest. AAW is not a diagnostic test or a measure of salience-network activation, vagal dominance, central autonomic network engagement, ketamine efficacy, treatment readiness, or clinical success. It is a pragmatic state-verification signal designed for a specific clinical question: Did the cardio-autonomic state move in the direction predicted by the Gate component of the framework under controlled measurement conditions? We use the term AAW descriptively rather than normatively: “affirmative” denotes concordance with the predicted cardio-autonomic direction, not clinical approval, treatment eligibility, or therapeutic success.

The need for this restraint follows from the nature of HRV itself. HRV is a useful but condition-sensitive measure. Standard references emphasize that HRV interpretation depends on measurement duration, physiological context, signal quality, and analytic choices (Circulation, 1996). Psychophysiological recommendations similarly stress careful control of experimental design, data analysis, and reporting when HRV or cardiac vagal tone is used (Laborde et al., 2017; Quintana and Heathers, 2014; Quintana et al., 2016). RMSSD is widely used as a short-term time-domain HRV measure and is often interpreted as reflecting vagally mediated beat-to-beat variability, but it remains sensitive to recording conditions, artifact, respiration, posture, medications, and clinical state (Shaffer and Ginsberg, 2017). For these reasons, AAW should never be interpreted outside its measurement constraints.

The term Window is deliberate. AAW is not a trait. It is not a patient category. It is not a general autonomic phenotype. It is a brief session-level pattern observed within a specified measurement window. A patient may show the AAW pattern in one session and not show it in another. This variability is not a weakness of the concept; it is the reason the concept is state-first.

Two AAW uses must be distinguished. AAW quality assurance (AAW-QA) refers to a quality-assurance or state-verification readout obtained after the clinical sequence has occurred. In the existing clinical anchor, AAW-QA is used retrospectively or observationally to ask whether a completed workflow was associated with the predicted cardio-autonomic direction. AAW-QA can motivate the Gate hypothesis, but it cannot prove prospective Gate readiness. AAW-Gate refers to a future pre-dose criterion. It would be assessed before ketamine administration to determine whether the patient has entered a cardio-autonomic state compatible with the Gate. AAW-Gate is not established by the existing cohort. It is a prospective hypothesis.

The paired nature of AAW is also important. Heart rate reduction alone is not sufficient. A lower heart rate may reflect rest, sedation, reduced pain, respiratory change, medication effect, fatigue, or measurement artifact. RMSSD increase alone is not sufficient either; RMSSD can be affected by respiration, signal artifact, ectopy, and analytic window length. The combined directional pattern - HR down and RMSSD up - does not solve these problems, but it creates a stricter and more interpretable candidate signal than either component alone.

Autonomic Affirmative Window also should not be treated as a surrogate outcome. It is not a replacement for patient-reported outcomes, behavioral outcomes, safety outcomes, or validated clinical measures. Its role is closer to a process-quality signal: a candidate indicator that the autonomic side of the Gate moved in the predicted direction under specified measurement conditions. Its value lies in testing whether a predicted cardio-autonomic shift occurred, not in identifying salience-network activation, determining treatment readiness, or predicting clinical success. A session showing the AAW pattern may still fail clinically if the patient cannot reintegrate. A session without the AAW pattern may still improve through non-autonomic pathways such as analgesia, expectancy, clinician support, local procedural effects, or spontaneous adaptation.

## Clinical anchor as observational provenance, not comparative evidence

The Gate-Amplifier-Reintegration framework is informed, but not validated, by clinical observations from a single-center outpatient chronic pain care pathway. The pathway consisted of consecutive registry cases from a routine-care program (*N* = 160; 1,490 ketamine sessions) in which awake low-dose ketamine was delivered under operational invariants and cervicothoracic sympathetic modulation could be timed before ketamine when clinically indicated as part of pain/autonomic care.

The clinical anchor is summarized concisely in Table 1 and in detail in Supplementary Data Sheet 1. It is included to document the observational provenance of the framework, not to estimate efficacy or provide comparative evidence for any route-defined workflow. The supplement provides the cohort definition, route categories, block and ketamine workflow definitions, chart-documented induction-improvement rule, observed dosing summary, patient-level safety summary, HRV quality-assurance window definition, artifact rule, and AAW-QA descriptive contrast.

**TABLE 1:** Clinical-anchor provenance and interpretive boundaries.

Domain	Descriptive information	Interpretive boundary
Cohort	Single-center outpatient chronic pain pathway; *N* = 160 patients; 1,490 ketamine sessions; 1,411 IV sessions and 79 non-IV sessions.	Observational provenance only; not an effectiveness trial.
Analytic sets	IV-dominant route-classified set *n* = 149; tie-excluded *n* = 8; no-IV patients *n* = 3.	Route classification was descriptive and nonrandomized.
Route categories	IV + Upper *n* = 57; IV-only *n* = 62; IV + Lower *n* = 12; IV + SGB *n* = 18.	Route groups are not exchangeable comparison groups.
Baseline imbalance	IV + Upper and IV-only differed in age, indication mix, post-2022 era, sessions per patient, and evaluable ratings.	These imbalances preclude causal or comparative interpretation.
Workflow	Awake low-dose ketamine under operational invariants; cervicothoracic sympathetic modulation could be timed before ketamine when clinically indicated.	Cervicothoracic modulation is one candidate Gate lever, not the theory itself.
Outcome definition	Chart-documented induction improvement within the first ≤ 6 rated IV sessions; responder-like summary defined as ≥ 80% improved among evaluable ratings.	Chart-based, unblinded, not a validated patient-reported outcome; groups differed in number of evaluable ratings.
Dosing	Observed induction dose was clinician-selected mg/kg within the induction window.	Process variable only; not biological dose requirement or dose-sparing evidence.
HRV-QA availability	Upper HRV-QA subset: 42 sessions/24 patients; Lower HRV-QA subset: 24 sessions/16 patients.	Post-sequence quality-assurance stream; not prospective Gate evidence or mechanistic inference.
HRV constraints	Forearm photoplethysmography-derived beat-to-beat intervals; supine posture; non-paced respiration; 3- or 5-min windows; Kubios automatic correction; version not preserved.	HRV/AAW is condition-sensitive and hypothesis-generating only.
Safety abstraction	Recorded patient-level ketamine-session events: SpO2 < 90% 12/160, oxygen mask use 8/160, nausea/vomiting 2/160. No documented block-related procedural complications or technical failures were identified in the reviewed categories.	Routine-care chart abstraction; not generalizable safety proof, not rare-event exclusion, and absence of recorded events is not proof of general neuraxial safety.
Neural measurement	No concurrent fMRI, EEG, fNIRS, intracranial recording, or direct salience-network measurement.	Systems-neuroscience mechanism remains theoretical and requires prospective testing.

Quantitative observations from this pathway, including chart-documented improvement, clinician-selected dosing, patient-level safety summaries, and AAW-QA patterns, should be interpreted only as source observations that raised a testable question. Route selection was nonrandomized, outcomes were chart-based, dosing was clinician-selected, sessions can repeat within individuals, group denominators and evaluable ratings differed, and no direct neural measurement was obtained.

The key point is conceptual: routine-care observations raised a specific systems question about whether autonomic state should be measured and manipulated prospectively before awake low-dose ketamine. The clinical anchor cannot determine whether cervicothoracic sympathetic modulation caused improvement, reduced ketamine dose, improved safety, or engaged the salience network.

## Alternative accounts and separable predictions

The candidate Gate component of the framework must be separated from plausible non-Gate explanations. Several alternative accounts could produce the same descriptive clinical pattern: analgesia from local procedures, expectancy, clinician attention, environmental containment, workflow maturation in the post-2022 era, patient selection, treatment persistence, documentation bias, conservative dosing practice, respiratory or posture-related HRV change, and photoplethysmography (PPG)-derived interval artifact. These accounts are not nuisance objections; they are competing explanations that future studies should test directly.

Each alternative account makes separable predictions. An analgesia account predicts that pain relief or reduced pain threat, rather than autonomic Gate status, explains downstream cooperation and documentation. An expectancy or clinician-attention account predicts similar improvement after credible contextual or non-invasive preparation. A workflow-era account predicts that calendar period and protocol maturity account for route-defined differences. A treatment-persistence account predicts that patients receiving more sessions accumulate more opportunities for chart-documented improvement. A measurement-artifact account predicts that heart rate/RMSSD directional concordance disappears when respiration, posture, sensor quality, window length, and repeated-session dependence are controlled.

For this reason, the framework should be evaluated against active comparators and context controls rather than against no explanation. Prospective studies should measure pain relief, expectation, clinician contact, session number, calendar period, respiratory context, posture, oxygen use, and signal quality alongside AAW-Gate and reintegration outputs. If these variables explain the observed pattern better than autonomic state, the framework should be revised toward analgesic, expectancy, workflow-stabilization, or peripheral measurement accounts.

## Falsifiable predictions and assay-lite tests

The Gate-Amplifier-Reintegration framework is useful only if it can fail. It should therefore be treated as a testable systems-neuroscience hypothesis, not as a retrospective explanation for a favorable clinical pattern. The clinical provenance motivated the framework, but prospective tests must determine whether autonomic Gate manipulation actually changes cardio-autonomic state, whether that state relates to cortical control signals, whether competing explanations better account for the pattern, and whether any process improves session-level steerability or reintegration.

The key experimental move is to separate Gate manipulation, ketamine amplification, and reintegration output. In the retrospective clinical anchor, these elements were embedded in routine care. In future studies, they should be temporally and analytically separated. At minimum, a prospective session should include a pre-Gate baseline, a post-Gate/pre-ketamine window, a ketamine exposure window, and a post-ketamine reintegration window. This allows investigators to distinguish AAW-Gate from AAW-QA and to test whether autonomic change occurs before ketamine rather than only after the entire clinical sequence.

The first prediction is autonomic Gate reproducibility. If cervicothoracic sympathetic modulation, or any alternative autonomic preparation, functions as a Gate lever, then it should reproducibly shift short-window HRV in the predicted direction under strict measurement conditions. Specifically, a pre-dose AAW-Gate pattern - heart rate down and RMSSD up from baseline to post-Gate/pre-ketamine - should occur more frequently after active Gate manipulation than after control conditions. Failure to reproduce this pattern under adequate quality control would weaken the autonomic Gate claim.

The second prediction is state-to-cortex convergence. AAW-Gate should not be considered meaningful merely because HR and RMSSD move in a favorable direction. The stronger prediction is that prospectively defined AAW-Gate-positive sessions should be tested for convergence with candidate low-burden EEG/fNIRS and behavioral readouts of salience switching and task-control engagement. These markers need not prove salience-network activation, and they should not be described as definitive biomarkers. Examples include oddball or deviance-related responses, mid-frontal theta during control demands, task-related prefrontal fNIRS oxygenation, or simple interference-task performance (Cavanagh and Frank, 2014; Eisma et al., 2021; Polich, 2007; Pinti et al., 2020).

The third prediction is frontoparietal-control-compatible reintegration. A stable Gate should not merely reduce subjective distress or dissociative intensity. It should increase the usability of the amplified state. Therefore, AAW-Gate positivity should be associated with more complete reintegration outputs: coherent language, joint attention, task completion, decision capture, or next-day action specification. These outputs are process markers, not definitive clinical endpoints. They test whether the altered state was organized into goal-directed control rather than remaining an intense but unstructured experience.

The fourth prediction is moderation/mediation by autonomic shift. If the framework is correct, autonomic state shifts should at minimum moderate, and in sufficiently powered designs may partly mediate, the relationship between Gate manipulation and session-level steerability. A randomized or within-patient design should test whether delta HR and delta RMSSD from baseline to post-Gate/pre-ketamine moderate, and where appropriate mediate, any part of the relationship between Gate manipulation and downstream outcomes such as tolerability, protocol stability, cortical control markers, or reintegration outputs. If clinical-process differences appear without any measurable autonomic moderation or mediation, alternative explanations - analgesia, expectancy, contextual reassurance, clinician behavior, or procedural salience - become more plausible.

The fifth prediction is dose non-primacy. In this framework, dose is not ignored, but it is not the organizing principle. A prospective study should test whether state-stabilized low-dose ketamine produces better steerability than dose escalation in an unstable state. If dose alone explains tolerability, reintegration, and clinical-process outcomes after randomization, then the Gate-Amplifier distinction is weakened. Conversely, if stable Gate conditions allow lower-dose ketamine to remain usable and coherent, the Amplifier component gains support.

These tests can be low-burden physiological and behavioral assays (“assay-lite” tests). The goal is not to reproduce a full scanner-based mechanistic study in every outpatient clinic. The goal is to add enough physiology and task structure to make the hypothesis falsifiable. A minimal assay-lite battery could include short-window HRV with strict quality control, a brief oddball or deviance task, a brief cognitive-control or interference task, optional frontal/prefrontal fNIRS, clinician-rated protocol deviations, patient-reported dysphoria/tolerability, and structured reintegration outputs. Such a battery would not prove salience-network mechanism by itself, but it would identify whether the autonomic Gate component is moving toward or away from the predicted pattern.

Table 2 summarizes the candidate tests, supportive patterns, and falsifier conditions for the proposed framework.

**TABLE 2:** Falsifiable predictions of the Gate-Amplifier-Reintegration framework.

Prediction	Minimal test	Supportive pattern	Falsifier/alternative
Autonomic Gate reproducibility	Compare baseline to post-Gate/pre-ketamine HR/RMSSD under fixed quality control conditions.	Active Gate increases AAW-Gate frequency vs. comparator.	No AAW-Gate difference under adequate quality control.
State-to-cortex convergence	Compare prospectively defined AAW-Gate-positive vs. negative sessions with brief EEG/fNIRS or task readouts.	Convergent candidate control/switching readouts (e.g., deviance/P300, mid-frontal theta, prefrontal fNIRS).	HRV shift without cortical or behavioral convergence.
Reintegration	Assess structured language, joint-attention, task, decision, and next-day action outputs.	More coherent behavioral control outputs in Gate-stable sessions.	Intense experience without task completion or action capture.
Moderation/mediation	Use randomized or within-patient Gate manipulation; test moderation first, mediation if powered.	Autonomic shift moderates, and possibly mediates, protocol stability or reintegration.	Differences better explained by expectancy, context, analgesia, or procedural effects.
Dose non-primacy	Compare state-stabilized low-dose sessions with dose escalation/unstable-state sessions.	Low-dose ketamine remains steerable under stable Gate conditions.	Dose alone explains outcomes after randomization.
Unstable Gate boundary	Track high vigilance, dysphoria, respiratory instability, absent AAW-Gate, or protocol drift.	Unstable Gate predicts poor tolerability or failed reintegration.	Good reintegration despite adverse/absent Gate signals.
Context control	Use lower-level block, non-invasive autonomic preparation, delayed timing, contextual control, or randomized sequence.	Active Gate pattern is not explained by context alone.	Equivalent outcomes across context controls.
Safety/feasibility	Track adverse events, neuraxial complications, oxygen use, nausea, protocol failure, and staff burden.	Gate manipulation remains feasible without unacceptable burden.	Risk or burden outweighs steerability gains.

## Boundary conditions and risks

The Gate-Amplifier-Reintegration framework is intentionally bounded. It should not be read as a claim that cervicothoracic sympathetic modulation is necessary for ketamine therapy, that HRV can determine treatment eligibility, or that the clinical anchor proves salience-network mechanism. The framework proposes a testable control sequence - Gate, Amplifier, Reintegration - but each component carries interpretive and clinical limits.

The first boundary is mechanistic. The clinical anchor did not include concurrent fMRI, EEG, fNIRS, intracranial recording, or direct salience-network measurement. Therefore, all claims involving salience-network gain, default-mode loosening, frontoparietal-control-compatible reintegration, thalamo-cortical selectivity, and central autonomic network interaction remain theoretical. They are grounded in existing systems-neuroscience literature, but they are not demonstrated by the outpatient cohort. The cohort motivates the model; it does not verify the neural mechanism.

The second boundary is physiological. HRV is indirect and condition-sensitive. Heart rate and RMSSD can be influenced by posture, respiration, sensor placement, ectopy, movement artifact, pain, anxiety, nausea, oxygen use, medications, sleep deprivation, cardiovascular disease, age, and time of day. For this reason, AAW should not be treated as a biomarker, a diagnostic test, or a stand-alone decision rule. AAW is only interpretable as a bounded state-verification pattern under strict measurement invariants. If those invariants are violated, the session may remain clinically meaningful, but the physiological inference should be downgraded.

The third boundary is causal. The clinical anchor was nonrandomized. Route selection, block timing, ketamine dosing, and session management were determined clinically. This means that observed differences between IV + Upper, IV-only, IV + Lower, and other route-defined workflows may reflect patient selection, indication, pain distribution, procedural feasibility, clinician judgment, calendar era, patient expectation, charting practice, or unmeasured severity rather than the Gate itself. A retrospective route-defined pattern is not a causal estimate.

The fourth boundary is outcome ascertainment. Chart-documented improvement is not equivalent to a validated patient-reported outcome. It can be useful as a pragmatic program signal, especially in real-world clinical pathways, but it is vulnerable to documentation bias, clinician expectation, missingness, variable timing, and inconsistent patient language. The clinical anchor can justify prospective testing; it cannot substitute for standardized pain, function, mood, dissociation, tolerability, and follow-up measures.

The fifth boundary is dosing interpretation. Observed ketamine dose was clinician-selected. Lower observed dose in one workflow should not be described as biological dose reduction or dose-sparing efficacy. It may reflect clinician confidence, patient phenotype, route selection, workflow maturity, safety preference, or session-by-session judgment. In the present model, dose is a process variable. It becomes mechanistically interpretable only in prospective designs that control or randomize Gate state and ketamine exposure.

The sixth boundary is procedural risk. Cervicothoracic epidural sympathetic modulation is an invasive neuraxial procedure. It requires appropriate expertise, patient selection, sterile technique, monitoring, rescue capacity, and governance. The framework should not be interpreted as recommending neuraxial procedures for ketamine preparation in settings without appropriate anesthesiology training or procedural infrastructure. The model is autonomic Gate, not epidural required. Non-invasive autonomic preparation, breathing/posture protocols, environmental control, and other safer Gate levers should be explored where appropriate.

In the clinical-anchor source records, no documented block-related procedural complications or technical failures were identified in the reviewed categories; nevertheless, this observation is setting-specific and should not be generalized as neuraxial safety evidence.

The seventh boundary is diagnostic scope. This paper is anchored in an outpatient chronic pain care pathway. It does not establish diagnosis-specific efficacy for depression, PTSD, anxiety disorders, OCD, ADHD, ASD, dissociative disorders, psychosis-spectrum conditions, or other psychiatric indications. The model may eventually be tested across state-defined phenotypes, but diagnosis-by-diagnosis treatment claims would be premature. At this stage, the safest claim is state-based: autonomic and salience stability may matter when ketamine-induced flexibility is expected to remain steerable.

The eighth boundary concerns high-risk mental states. Patients with psychosis-spectrum vulnerability, severe dissociative instability, extreme threat vigilance, active mania, severe cognitive disorganization, or inability to participate in monitoring and reintegration may not be appropriate for a state-first awake ketamine protocol without additional safeguards. In such contexts, ketamine amplification could increase mis-salience, paranoia, fragmentation, dysphoria, or loss of behavioral steerability. These states should be treated as boundary conditions, not as routine extensions of the model.

These boundary conditions are not weaknesses to hide. They are part of the framework. A theory that specifies when it should not be applied is stronger than one that expands without limits. A positive HRV pattern should never override clinical judgment, patient distress, oxygen desaturation, procedural complication, inability to communicate, or failure to reintegrate. The Gate-Amplifier-Reintegration framework should therefore be advanced only as a falsifiable framework for prospective testing.

## Discussion: from dose-first ketamine to state-first control

This paper proposes the Gate-Amplifier-Reintegration framework for awake low-dose ketamine response. The framework is built around a simple control sequence: Gate, Amplifier, Reintegration. Ketamine is treated as an Amplifier of network flexibility, not as the upstream state condition proposed to shape therapeutic direction. The candidate Gate is autonomic-salience stability: a physiological and attentional state in which salience-mediated switching may remain more steerable during ketamine exposure. Reintegration is the process by which amplified flexibility is organized into language, joint attention, task context, decisions, and next-day action.

This distinction reframes ketamine treatment. In a dose-first view, the central question is how much ketamine is required to produce a clinical effect. In the Gate-Amplifier-Reintegration framework, the first question is different: What state is ketamine entering? If the patient enters ketamine exposure in a state of autonomic alarm, unstable vigilance, respiratory irregularity, pain threat, or interoceptive uncertainty, the drug may amplify that instability. If the patient enters in a more stable autonomic-salience state, the same pharmacologic amplification may be easier to guide toward frontoparietal-control-compatible reintegration.

The framework does not deny the importance of dose. Dose matters for pharmacologic exposure, safety, tolerability, and neurobiological effect. But dose alone is an incomplete organizing principle. Ketamine studies show effects on large-scale functional connectivity, including default-mode, salience, executive-control, thalamo-cortical, and prefrontal systems (Mueller et al., 2018; Fleming et al., 2019; Höflich et al., 2015; Zacharias et al., 2020; Moujaes et al., 2024). These effects are state-sensitive and variable across individuals. The present framework therefore treats dose as one component of a broader control problem, not as the sole determinant of therapeutic direction.

A central implication is that subjective intensity is not the same as therapeutic steerability. A highly intense ketamine session may be clinically unhelpful if it produces dysphoric dissociation, vigilance lability, disorganized salience, or failure of reintegration. Conversely, a less dramatic session may be more useful if it preserves wakefulness, language, joint attention, and actionable follow-through. The therapeutic target is not maximal dissociation or maximal phenomenological novelty. The target is steerable flexibility.

The roles of HRV and AAW are deliberately limited. AAW is not a biomarker of salience-network activation and should not be used as a stand-alone treatment decision rule. It is a bounded process-quality signal: a directional session-level pattern in which heart rate decreases and RMSSD increases under strict measurement conditions. This restraint is essential because HRV is condition-sensitive and indirect (Circulation, 1996; Laborde et al., 2017; Quintana and Heathers, 2014; Quintana et al., 2016). The value of AAW lies not in proving mechanism, but in providing a pragmatic way to test whether the autonomic side of the Gate moves in the predicted direction under controlled conditions.

The clinical provenance should be interpreted in the same restrained way. The outpatient chronic pain cohort describes how several observations appeared within the same real-world pathway: awake low-dose ketamine, routine cervicothoracic sympathetic modulation timed before ketamine, chart-documented improvement, observed dosing, patient-level safety monitoring, and an HRV quality-assurance pattern. This combination is useful for generating a question, but it is not comparative evidence. It does not show that cervicothoracic sympathetic modulation timed before ketamine caused improvement, that ketamine dose requirements were biologically reduced, or that salience-network switching occurred. The cohort is a disciplined source of framework generation, not the endpoint of the argument.

The strongest version of the framework will require prospective falsification. The first step is randomized or otherwise well-controlled Gate manipulation. The key pre-ketamine measure is AAW-Gate, not retrospective AAW-QA. AAW-Gate should be assessed after Gate manipulation and before ketamine exposure to determine whether the predicted cardio-autonomic shift occurred before the Amplifier was introduced. The next step is convergence with cortical and behavioral readouts: EEG oddball or deviance paradigms, mid-frontal theta during cognitive control, prefrontal fNIRS during task or reintegration phases, brief interference tasks, and structured language/action outputs.

The framework should also fail under defined conditions. If Gate manipulation does not change AAW-Gate under adequate measurement quality, the autonomic Gate claim weakens. If AAW-Gate changes but does not relate to cortical or behavioral control markers, AAW may be peripheral or epiphenomenal. If intense subjective ketamine experiences repeatedly produce poor reintegration despite favorable physiology, subjective intensity should be deprioritized as a target. If patients show good outcomes despite absent or adverse Gate signals, the Gate-first requirement is too strong and must be revised.

These falsifiers and alternative accounts are not secondary caveats. They are central to the framework. The Gate-Amplifier-Reintegration framework becomes scientifically useful only if it can specify what evidence would weaken it and what non-Gate explanations would supersede it. The aim is not to defend a preferred procedure or to relabel a clinical observation with network terminology. The aim is to convert a clinical observation into a testable systems-neuroscience program. The next step is not stronger rhetoric; it is prospective falsification.

## Conclusion

We propose the Gate-Amplifier-Reintegration framework for awake low-dose ketamine response. Ketamine is the Amplifier, not the Gate. The candidate Gate is autonomic-salience stability, and Reintegration is the organization of amplified flexibility into language, joint attention, task context, and action. A single-center outpatient chronic pain pathway provides observational provenance for the framework, but not causal, comparative, or mechanistic proof. This framework should not be interpreted as a recommendation for neuraxial preparation before ketamine; procedural Gate levers should be tested only with appropriate expertise and governance, and alongside non-invasive autonomic preparation strategies. It should now be tested prospectively: manipulate the Gate, measure AAW-Gate before ketamine, assess cortical and behavioral convergence, evaluate competing explanations, and determine whether autonomic shifts moderate or mediate tolerability and usable clinical change.

## Data Availability

The datasets presented in this article are not readily available because the underlying individual-level and session-level datasets are not publicly available and are not available as raw datasets by request because they derive from single-center routine clinical care records and may contain potentially re-identifiable clinical information even after de-identification. De-identified aggregate summaries supporting the clinical-anchor description are provided in the article and Supplementary Data Sheet 1. Selected additional de-identified summary materials may be considered upon reasonable request, subject to institutional approval, ethics requirements, and data protection constraints. Requests to access the datasets should be directed to KT, torii@tokyomasui.jp.

## Supplementary material

Supplementary Data Sheet 1 Clinical-anchor methods, cohort denominators, route definitions, HRV-QA/AAW-QA measurement constraints, safety abstraction, and interpretive boundaries for the single-center outpatient chronic pain pathway.

## References

[B1] Beissner F. Meissner K. Bär K. J. Napadow V. (2013). The autonomic brain: An activation likelihood estimation meta-analysis for central processing of autonomic function. *J. Neurosci.* 33 10503–10511. 10.1523/JNEUROSCI.1103-13.2013 23785162 PMC3685840

[B2] Borsook D. Edwards R. Elman I. Becerra L. Levine J. (2013). Pain and analgesia: The value of salience circuits. *Prog. Neurobiol.* 104 93–105. 10.1016/j.pneurobio.2013.02.003 23499729 PMC3644802

[B3] Caruana N. Brock J. Woolgar A. (2015). A frontotemporoparietal network common to initiating and responding to joint attention bids. *Neuroimage* 108 34–46. 10.1016/j.neuroimage.2014.12.041 25534111

[B4] Cavanagh J. F. Frank M. J. (2014). Frontal theta as a mechanism for cognitive control. *Trends Cogn. Sci.* 18 414–421. 10.1016/j.tics.2014.04.007 24835663 PMC4112145

[B5] Circulation (1996). Heart rate variability: Standards of measurement, physiological interpretation and clinical use task force of the European society of cardiology and the North American society of pacing and electrophysiology. *Circulation* 93 1043–1065. 10.1161/01.CIR.93.5.1043 8598068

[B6] Cohen S. P. Bhatia A. Buvanendran A. Schwenk E. S. Wasan A. D. Hurley R. W. et al. (2018). Consensus guidelines on the use of intravenous ketamine infusions for chronic pain from ASRA, AAPM, and ASA. *Reg. Anesth. Pain Med.* 43 521–546. 10.1097/AAP.0000000000000808 29870458 PMC6023575

[B7] Cole M. W. Reynolds J. R. Power J. D. Repovs G. Anticevic A. Braver T. S. (2013). Multi-task connectivity reveals flexible hubs for adaptive task control. *Nat. Neurosci.* 16 1348–1355. 10.1038/nn.3470 23892552 PMC3758404

[B8] Critchley H. D. Wiens S. Rotshtein P. Öhman A. Dolan R. J. (2004). Neural systems supporting interoceptive awareness. *Nat. Neurosci.* 7 189–195. 10.1038/nn1176 14730305

[B9] Eisma J. Rawls E. Long S. Mach R. Lamm C. (2021). Frontal midline theta differentiates separate cognitive control strategies while still generalizing the need for cognitive control. *Sci. Rep.* 11:14641. 10.1038/s41598-021-94162-z 34282209 PMC8290013

[B10] Fedorenko E. Thompson-Schill S. L. (2014). Reworking the language network. *Trends Cogn. Sci.* 18 120–126. 10.1016/j.tics.2013.12.006 24440115 PMC4091770

[B11] Ferraro S. Klugah-Brown B. Tench C. R. Bazinet V. Bore M. C. Nigri A. et al. (2022). The central autonomic system revisited: Convergent evidence for a regulatory role of the insular and midcingulate cortex from neuroimaging meta-analyses. *Neurosci. Biobehav. Rev.* 142:104915. 10.1016/j.neubiorev.2022.104915 36244505

[B12] Fleming L. M. Javitt D. C. Carter C. S. Kantrowitz J. T. Girgis R. R. Kegeles L. S. et al. (2019). A multicenter study of ketamine effects on functional connectivity: Large scale network relationships, hubs and symptom mechanisms. *Neuroimage Clin.* 22:101739. 10.1016/j.nicl.2019.101739 30852397 PMC6411494

[B13] Höflich A. Hahn A. Küblböck M. Kranz G. S. Vanicek T. Windischberger C. et al. (2015). Ketamine-induced modulation of the thalamo-cortical network in healthy volunteers as a model for schizophrenia. *Int. J. Neuropsychopharmacol.* 18:yv040. 10.1093/ijnp/pyv040 25896256 PMC4576520

[B14] Johansson E. Xiong H.-Y. Polli A. Coppieters I. Nijs J. (2024). Towards a real-life understanding of the altered functional behaviour of the default mode and salience network in chronic pain: Are people with chronic pain overthinking the meaning of their pain? *J. Clin. Med.* 13:1645. 10.3390/jcm13061645 38541870 PMC10971341

[B15] Laborde S. Mosley E. Thayer J. F. (2017). Heart rate variability and cardiac vagal tone in psychophysiological research: Recommendations for experiment planning, data analysis, and data reporting. *Front. Psychol.* 8:213. 10.3389/fpsyg.2017.00213 28265249 PMC5316555

[B16] Menon V. Uddin L. Q. (2010). Saliency, switching, attention and control: A network model of insula function. *Brain Struct. Funct.* 214 655–667. 10.1007/s00429-010-0262-0 20512370 PMC2899886

[B17] Moujaes F. Ji J. L. Rahmati M. Murray J. D. Repovs G. Anticevic A. (2024). Ketamine induces multiple individually distinct whole-brain functional connectivity signatures. *eLife* 13:e84173. 10.7554/eLife.84173 38629811 PMC11023699

[B18] Mueller F. Musso F. London M. de Boer P. Zacharias N. Winterer G. (2018). Pharmacological fMRI: Effects of subanesthetic ketamine on resting-state functional connectivity in the default mode network, salience network, dorsal attention network and executive control network. *Neuroimage Clin.* 19 745–757. 10.1016/j.nicl.2018.05.037 30003027 PMC6040604

[B19] Mundy P. Newell L. (2007). Attention, joint attention, and social cognition. *Curr. Dir. Psychol. Sci.* 16 269–274. 10.1111/j.1467-8721.2007.00518.x 19343102 PMC2663908

[B20] Pinti P. Tachtsidis I. Hamilton A. Hirsch J. Aichelburg C. Gilbert S. et al. (2020). The present and future use of functional near-infrared spectroscopy for cognitive neuroscience. *Ann. N. Y. Acad. Sci.* 1464 5–29. 10.1111/nyas.13948 30085354 PMC6367070

[B21] Polich J. (2007). Updating P300: An integrative theory of P3a and P3b. *Clin. Neurophysiol.* 118 2128–2148. 10.1016/j.clinph.2007.04.019 17573239 PMC2715154

[B22] Quintana D. S. Alvares G. A. Heathers J. A. J. (2016). Guidelines for reporting articles on psychiatry and heart rate variability (GRAPH): Recommendations to advance research communication. *Transl. Psychiatry* 6:e803. 10.1038/tp.2016.73 27163204 PMC5070064

[B23] Quintana D. S. Heathers J. A. J. (2014). Considerations in the assessment of heart rate variability in biobehavioral research. *Front. Psychol.* 5:805. 10.3389/fpsyg.2014.00805 25101047 PMC4106423

[B24] Raja S. N. Carr D. B. Cohen M. Finnerup N. B. Flor H. Gibson S. et al. (2020). The revised international association for the study of pain definition of pain: Concepts, challenges, and compromises. *Pain* 161 1976–1982. 10.1097/j.pain.0000000000001939 32694387 PMC7680716

[B25] Schwenk E. S. Viscusi E. R. Buvanendran A. Hurley R. W. Wasan A. D. Narouze S. et al. (2018). Consensus guidelines on the use of intravenous ketamine infusions for acute pain management from ASRA, AAPM, and ASA. *Reg. Anesth. Pain Med.* 43 456–466. 10.1097/AAP.0000000000000806 29870457 PMC6023582

[B26] Seeley W. W. Menon V. Schatzberg A. F. Keller J. Glover G. H. Kenna H. et al. (2007). Dissociable intrinsic connectivity networks for salience processing and executive control. *J. Neurosci.* 27 2349–2356. 10.1523/JNEUROSCI.5587-06.2007 17329432 PMC2680293

[B27] Seth A. K. (2013). Interoceptive inference, emotion, and the embodied self. *Trends Cogn. Sci.* 17 565–573. 10.1016/j.tics.2013.09.007 24126130

[B28] Seth A. K. Friston K. J. (2016). Active interoceptive inference and the emotional brain. *Philos. Trans. R. Soc. Lond. B Biol. Sci.* 371:20160007. 10.1098/rstb.2016.0007 28080966 PMC5062097

[B29] Shaffer F. Ginsberg J. P. (2017). An overview of heart rate variability metrics and norms. *Front. Public Health* 5:258. 10.3389/fpubh.2017.00258 29034226 PMC5624990

[B30] Sridharan D. Levitin D. J. Menon V. (2008). A critical role for the right fronto-insular cortex in switching between central-executive and default-mode networks. *Proc. Natl. Acad. Sci. U. S. A.* 105 12569–12574. 10.1073/pnas.0800005105 18723676 PMC2527952

[B31] Thayer J. F. Lane R. D. (2000). A model of neurovisceral integration in emotion regulation and dysregulation. *J. Affect. Disord.* 61 201–216. 10.1016/S0165-0327(00)00338-4 11163422

[B32] Treede R.-D. Rief W. Barke A. Aziz Q. Bennett M. I. Benoliel R. et al. (2015). A classification of chronic pain for ICD-11. *Pain* 156 1003–1007. 10.1097/j.pain.0000000000000160 25844555 PMC4450869

[B33] van Ettinger-Veenstra H. Boehme R. Ghazanfari N. Olausson H. Wicksell R. K. Gerdle B. (2019). Chronic widespread pain patients show disrupted cortical connectivity in default mode and salience networks, modulated by pain sensitivity. *J. Pain Res.* 12 1743–1755. 10.2147/JPR.S189443 31213886 PMC6549756

[B34] Waskom M. L. Kumaran D. Gordon A. M. Rissman J. Wagner A. D. (2014). Frontoparietal representations of task context support the flexible control of goal-directed cognition. *J. Neurosci.* 34 10743–10755. 10.1523/JNEUROSCI.5282-13.2014 25100605 PMC4200112

[B35] Zacharias N. Musso F. Müller F. Lammers F. Saleh A. London M. et al. (2020). Ketamine effects on default mode network activity and vigilance: A randomized, placebo-controlled crossover simultaneous fMRI/EEG study. *Hum. Brain Mapp.* 41 107–119. 10.1002/hbm.24791 31532029 PMC7268043

[B36] Zanto T. P. Gazzaley A. (2013). Fronto-parietal network: Flexible hub of cognitive control. *Trends Cogn. Sci.* 17 602–603. 10.1016/j.tics.2013.10.001 24129332 PMC3873155

