# Supplementary material for: Autonomic-salience stability as a candidate Gate for awake low-dose ketamine: a systems neuroscience framework with a clinical anchor
Source: Front Syst Neurosci. 2026 Jul 1;20:1880737. doi: 10.3389/fnsys.2026.1880737 (PMC13368987; doi:10.3389/fnsys.2026.1880737)
Supplement: Supplementary Data Sheet 1 — Clinical-anchor methods, cohort denominators, route definitions, HRV-QA/AAW-QA measurement constraints, safety abstraction, and interpretive boundaries for the single-center outpatient chronic pain pathway. [file Data_Sheet_1.DOCX]

# Supplementary Data Sheet 1. Clinical anchor methods, definitions, denominator transparency, and descriptive summary

This supplementary data sheet documents the clinical-anchor dataset used only to describe the observational provenance of the Gate-Amplifier-Reintegration framework. It is not an effectiveness analysis and should not be interpreted as causal, comparative, or mechanistic evidence. Route-specific summaries are provided for denominator transparency and hypothesis generation only. In this supplement, HRV-QA means HRV quality assurance, and AAW-QA means Autonomic Affirmative Window quality assurance.

## 1. Data freezes and source records

Clinical endpoints and patient-level safety abstraction were frozen at the June 2025 clinical data freeze under protocol 005. The prospective HRV quality-assurance sub-study (protocol 004) continued collection through November 2025; HRV results are therefore treated as a QA/state-verification stream and do not update the frozen clinical endpoint counts. Source records included routine anesthesia records, procedure notes, ketamine dosing logs, vital sign monitoring records, and post-session clinician assessments.

## Supplementary Table S1. Cohort and analytic sets

| **Item** | **Definition / value** |
| --- | --- |
| Cohort | Consecutive registry cases 1–160 from a single outpatient anesthesiology chronic pain care pathway in Nagoya, Japan; N=160 patients. |
| Inclusion | Adults (>=18 years) in the chronic pain care pathway who received at least one ketamine treatment session (IV or IM) or were retained in the frozen care-pathway registry denominator. |
| Excluded pathway | Separate psychiatric ketamine protocols in the same institutional database were excluded because indication, dosing, and supportive care differed substantially. |
| Full descriptive cohort | All 160 patients retained for session-volume summaries and patient-level safety summaries. |
| IV-dominant route-classified set | Patients with >=1 IV session assignable to a single dominant IV route component using the largest count of each patient’s IV sessions; ties excluded a priori; n=149. |
| No-IV patients | Three patients without IV sessions were excluded from IV-dominant route comparisons and retained only for descriptive context (2 IM-only; 1 no IV session recorded in the frozen route data). |
| Session volume | 1,490 ketamine sessions total; 1,411 IV sessions and 79 non-IV sessions in the clinical data freeze. |

## Supplementary Table S2. Route-category denominators and induction evaluability

Induction evaluability here means at least one evaluable rated session within the first <=6 rated IV sessions for IV-route categories. Indeterminate/other session-level ratings were excluded from the session denominator by rule, but session-level counts of such ratings were not preserved in the patient-level master file.

| **Route category** | **n patients** | **Induction-evaluable patients** | **Non-evaluable patients** | **Responder count** | **Reason / boundary** |
| --- | --- | --- | --- | --- | --- |
| IV + Upper | 57 | 57 | 0 | 48 | Dominant IV route |
| IV-only | 62 | 62 | 0 | 25 | Dominant IV route |
| IV + Lower | 12 | 12 | 0 | 6 | Dominant IV route |
| IV + SGB | 18 | 18 | 0 | 12 | Dominant IV route |
| Tie-excluded | 8 | 8 | 0 | 4 | Equal largest IV-route counts; excluded from IV-dominant route-classified set |
| No IV sessions | 3 | NA | NA | NA | No IV sessions; excluded from IV-dominant route-classified set |

## Supplementary Table S3. Route-stratified baseline descriptors

Baseline descriptors are provided to make selection and era imbalance visible, not to adjust or estimate treatment effects. Indication categories use routine chart abstraction within the chronic pain pathway.

| **Route** | **n** | **Age, median [IQR]** | **Female** | **Indication categories, n** | **Post-2022 era** | **Sessions/patient, median [IQR]** | **Evaluable ratings, median [IQR]** |
| --- | --- | --- | --- | --- | --- | --- | --- |
| IV + Upper | 57 | 47.0 [38.0-56.0] | 36/57 (63.2%) | Pain only 10; Pain+psych 45; Psych-pred 2; Other/unclear 0 | 29/57 (50.9%) | 6.0 [2.0-14.0] | 6.0 [2.0-6.0] |
| IV-only | 62 | 54.5 [42.5-67.0] | 32/62 (51.6%) | Pain only 28; Pain+psych 31; Psych-pred 2; Other/unclear 1 | 15/62 (24.2%) | 1.0 [1.0-4.0] | 1.0 [1.0-4.0] |
| IV + Lower | 12 | 47.5 [39.0-68.0] | 7/12 (58.3%) | Pain only 4; Pain+psych 8; Psych-pred 0; Other/unclear 0 | 3/12 (25.0%) | 2.0 [1.0-5.0] | 2.0 [1.0-4.5] |
| IV + SGB | 18 | 55.0 [39.5-60.8] | 9/18 (50.0%) | Pain only 5; Pain+psych 13; Psych-pred 0; Other/unclear 0 | 4/18 (22.2%) | 3.0 [1.0-9.5] | 3.0 [1.0-6.0] |
| Tie-excluded | 8 | 60.5 [49.0-68.0] | 4/8 (50.0%) | Pain only 4; Pain+psych 4; Psych-pred 0; Other/unclear 0 | 1/8 (12.5%) | 2.0 [2.0-4.2] | 2.0 [2.0-3.8] |
| No IV sessions | 3 | 45.5 [38.8-52.2] | 1/3 (33.3%) | Pain only 0; Pain+psych 2; Psych-pred 1; Other/unclear 0 | 2/3 (66.7%) | 2.0 [1.5-2.5] | NA |

## 2. Route and workflow definitions

| **Item** | **Definition / value** |
| --- | --- |
| Awake low-dose ketamine invariants | No sedative premedication, supine posture, room-air breathing by default unless clinically indicated, slow continuous IV infusion without ketamine bolus, mid-infusion escalation generally avoided, continuous pulse oximetry/heart-rate monitoring, intermittent blood pressure checks, and discharge criteria. |
| IV-only | IV ketamine session without neuraxial block. |
| IV + Upper | IV ketamine with high-thoracic/low-cervical epidural sympathetic modulation timed before ketamine in the same session; anatomical range C7/T1–T4. |
| IV + Lower | IV ketamine with lower/lumbar epidural sympathetic modulation timed before ketamine in the same session. |
| IV + SGB | IV ketamine with stellate ganglion block route component. |
| Epidural technique | Paramedian single-shot technique with loss of resistance to saline, performed by an experienced anesthesiologist as routine pain/autonomic care. Local infiltration used 0.5% mepivacaine approximately 1.5 mL. Upper used 0.2% mepivacaine 5 mL at C7/T1–T4; lower used 0.2% mepivacaine 5 mL at lower/lumbar levels. |
| Test-dose practice | Epinephrine-containing test doses were not part of routine practice in this program. Safety was supported by slow incremental injection, repeated negative aspiration, continuous verbal monitoring, and standard physiological monitoring. This description is provided for transparency and is not a recommendation to omit test doses in other settings. |

## 3. Chart-documented induction-improvement and dosing definitions

| **Item** | **Definition / value** |
| --- | --- |
| Induction window | First <=6 rated IV sessions in chronological order. |
| Session rating | Routine chart documentation coded as Improved, Not improved, or Indeterminate/other using a hierarchical source rule: same-day pre-discharge clinician rating when available; otherwise the first subsequent clinic note explicitly attributing improvement/non-improvement to the index session. |
| Induction-phase responder classification | Patient-level summary defined as >=80% Improved among evaluable rated IV sessions within the induction window; Indeterminate/other ratings were non-evaluable and excluded from the denominator. |
| Observed induction dose | Session-level dose (mg/kg) = total IV ketamine amount per session divided by recorded body weight. Minimum induction dose is the lowest mg/kg within the first <=6 rated IV sessions. This is clinician-selected observed dosing, not biological dose requirement or dose-sparing evidence. |

## 4. Descriptive clinical-anchor summary and procedure-specific safety abstraction

| **Domain** | **Descriptive observation** | **Interpretive boundary** |
| --- | --- | --- |
| Session volume | N=160 patients; 1,490 ketamine sessions total; 1,411 IV sessions; 79 non-IV sessions. | Feasibility/context only. |
| Route composition of IV sessions | IV + Upper 674/1,411 (47.8%); IV-only 415/1,411 (29.4%); IV + Lower 140/1,411 (9.9%); IV + SGB 182/1,411 (12.9%). | Session distribution, not outcome evidence. |
| Chart-documented induction-improvement summary | In the IV-dominant set, IV + Upper 48/57 (84.2%) and IV-only 25/62 (40.3%). | Nonrandomized, chart-based, clinician-documented, and not a validated PRO endpoint. |
| Observed induction dose | Median minimum induction dose: IV + Upper 0.1538 [0.1111–0.2000] mg/kg; IV-only 0.2000 [0.1395–0.2431] mg/kg. | Clinician-selected process variable; not dose requirement or dose-sparing proof. |
| Recorded patient-level ketamine-session safety context | Full-cohort events: SpO2 <90% 12/160; oxygen mask use 8/160; nausea/vomiting 2/160. | Routine-care safety summary; not generalizable safety proof or rare-event exclusion. |
| Procedure-specific neuraxial safety abstraction | No documented block-related procedural complications or technical failures were identified in source records in the reviewed categories: accidental dural puncture, suspected intravascular injection, local anesthetic systemic toxicity, new neurologic deficit, infection, hematoma, prolonged/unexpected block requiring intervention, hypotension/bradycardia requiring escalation, unplanned transfer, emergency response, or delayed procedural complication. | Setting-specific chart abstraction; absence of recorded events is not proof of general neuraxial safety. |

## 5. HRV quality-assurance window, artifact rule, and subset denominators

HRV was recorded using Polar Verity Sense forearm photoplethysmography-derived beat-to-beat interval data with the patient supine and breathing gently without paced respiration. Respiratory rate output was available when recorded, but respiration was not paced or experimentally controlled. Two windows were analyzed per session: T1 (post-procedural modulation baseline for modulated sessions or pre-ketamine for ketamine-only sessions) and T2 (pre-discharge after ketamine). Each window was 3 or 5 minutes. HRV data were processed in Kubios HRV Scientific with automatic beat correction (medium); the software version was not preserved in the frozen HRV source sheet. If artifact exceeded 5% on the first attempt, one immediate re-measurement was obtained; recordings with >5% artifact after re-measurement were excluded. The prespecified AAW-QA pattern was delta HR <0 and delta RMSSD >0 from T1 to T2 under the same posture and device mount. Respiration, pain relief, anxiety, medications, oxygen use, movement, and repeated-session dependence were not experimentally controlled; therefore, these factors remain potential explanations of HR/RMSSD change rather than nuisance variables already removed. Because sessions can repeat within individuals, HRV contrasts are descriptive quality-assurance signals rather than mechanistic inference.

| **HRV-QA contrast subset** | **Sessions** | **AAW-QA positive sessions** | **Unique patients** | **Sessions per patient, median (range)** |
| --- | --- | --- | --- | --- |
| Upper prespecified band | 42 | 24 | 24 | 1.5 (1-4) |
| Lower prespecified band | 24 | 5 | 16 | 1.0 (1-3) |

The Upper vs Lower HRV-QA contrast used prespecified anatomical bands: Upper included C7/T1 and T3/4 sessions; Lower included lower/lumbar comparators including T12/L1 and lumbar levels. Other thoracic levels were not included in this Upper-vs-Lower contrast. The contrast was chosen because both categories involve epidural modulation and therefore provide a route-topography comparison; it should not be interpreted as a mechanistic or causal comparison.

## 6. Statistical and interpretive boundaries

The clinical anchor is descriptive: route assignment was nonrandomized, charting was unblinded, standardized PROs were not used, repeated HRV sessions were not modeled, and era/indication/session-count differences could confound route contrasts. Original risk differences, p-values, and sensitivity analyses are not used in the main manuscript as efficacy evidence, and the route-specific summaries should not be read as comparative estimates. Because the number of evaluable ratings differed substantially across route categories, the induction-phase responder summary should not be interpreted as an exchangeable comparative endpoint. A patient with one evaluable rating and a patient with several evaluable ratings do not contribute equivalent evidence to the >=80% Improved rule. These summaries are therefore retained only as descriptive provenance for hypothesis generation.
